# Genome-Wide Identification and Expression Analysis of Carotenoid Cleavage Dioxygenase Genes in *Salvia miltiorrhiza*

**DOI:** 10.3390/ijms252313138

**Published:** 2024-12-06

**Authors:** Minyu Shou, Qinzhe Lin, Lulu Peng, Zijie Wang, Ying Xu, Jiaochen Qi, Degang Zhao, Min Shi, Guoyin Kai

**Affiliations:** 1 Laboratory of Medicinal Plant Biotechnology, School of Pharmaceutical Sciences, Zhejiang Chinese Medical University, Hangzhou 310053, China; smy252900@126.com (M.S.); 13250866727@163.com (Q.L.); 15290280761@163.com (L.P.); wangzijie120719@163.com (Z.W.); 13819964762@163.com (Y.X.); 15990075094@163.com (J.Q.); 2 The Key Laboratory of Plant Resources Conservation and Germplasm Innovation in Mountainous Region, Ministry of Education, Institute of Agro-Bioengineering, College of Life Sciences, Guizhou University, Guiyang 550025, China; dgzhao@gzu.edu.cn

**Keywords:** *Salvia miltiorrhiza*, carotenoid cleavage dioxygenases, genome-wide identification, hormone response

## Abstract

In the process of catalyzing carotenoids into various apocarotenoids and other derivatives, carotenoid cleavage dioxygenases (CCDs) play key roles. However, little information on CCDs has been reported in regard to *Salvia miltiorrhiza*. In this study, a total of 21 CCD genes were identified in the whole genome of *S. miltiorrhiza*, mainly distributed between five chromosomes. Phylogenetic relationship analysis revealed that 21 SmCCD genes were classified into four subfamilies, including SmCCD4, 7, 8, and NCED; the members of the same subfamily show similar gene structures and tertiary structures. The interspecific collinearity with other plant species, such as *Arabidopsis thaliana* and *Oryza sativa* was analyzed. *Cis*-elements analysis demonstrated that the majority were stress response-, light response-, growth-, and development-related. The expression pattern of the SmCCD genes was expressed in the analyzed tissues. Furthermore, the majority of the SmCCD4 subfamily members varied in their expression levels under the treatment of MeJA, YE, and ABA, indicating the potential function of SmCCD4 in the metabolism process of *S. miltiorrhiza*. In general, this study provides a systematic analysis of SmCCD genes and lays the foundation for uncovering the regulation and function of SmCCD genes in *S. miltiorrhiza*.

## 1. Introduction

Widely abundant natural pigments, there are more than 700 types of carotenoids on earth [1]. Carotenoid pigments are mainly considered to be C_40_ lipophilic isoprenoids, synthesized in all photosynthetic organisms (bacteria, algae, and plants), while some non-photosynthetic bacteria and fungi also have such an ability [2]. Among them, plant carotenoids are tetraterpenes derived from the C_40_ isoprenoid phytoene, and hundreds of plant carotenoids can be classified into two major sorts, carotenes and xanthophylls [3]. Moreover, carotenoids are far more than just normal pigments, also playing important roles in the process of photosynthesis, plant signaling, plant development, and so on [4,5]. Carotenoid cleavage dioxygenases (CCDs) have the ability to mediate the specific cleavage of carotenoids to generate different apocarotenoids and their derivatives [6]. Based on their characteristics of promoting substrate epoxidation, the CCD members of plants are sorted into two main subfamilies called CCD and NCED (9-cisepoxycarotenoid dioxygenase) [7]. Compared to the catalysis specificity of NCED, CCD enzymes do not have the specific cleavage sites of substrates—cleave carotenoid and apocarotenoid substrates—while NCEDs can form xanthoxin by catalyzing violaxanthin or neoxanthin, which is considered a rate-limiting step in ABA biosynthesis [8]. For example, AtCCD7 has been proven to perform the primary cleavage of C_40_ to C_27_ in the plastid; C_27_, as a major substrate for AtCCD1, is then able to develop further cleavage in cytosol [9]. It is likely that sequential cleavages of AtCCD7 and AtCCD8 to β-carotene are the initial steps in the synthesis of a carotenoid-derived signaling [10]. When it comes to CCD4, only one CCD4 has been identified in *Arabidopsis thaliana,* although CCD4 members are considered complex, an in vitro study suggested that AtCCD4 cleaves all-trans-bicyclic carotenoids [11], and its function and identification have been explored in *Crocus sativus*, peaches, and potatoes [12,13,14].

The CCD gene family is ancient, and the first carotenoid cleavage dioxygenase was found in the maize mutant *vp14* [8]. Commonly, CCD members share the conserved retinal pigment epithelial membrane protein (RPE65) domain. Four His and three Glu (or less commonly Asp) residues decide the sequence feature of CCDs, and these residues always exist in the location of high sequence conservation, which proves the homology of CCDs [15]. Furthermore, according to the evolutionary origin excavation of 158 CCD genes in more than ten plant species, two groups were sorted. Group 1 comprised CCD7 and CCD8, each with unique independent evolutionary origins, respectively, and group 2 was divided into NCED, CCD-like, CCD4, and CCD1, which are speculated to derive from their common ancestor [16]. In addition, the CCD genes could be directly grouped into five classes including CCD1, 4, 7, 8, and NCED. For example, nine CCD genes have been identified in *A. thaliana*, consisting of four CCDs (CCD1, 4, 7, and 8) and five NCEDs (NCED2, 3, 5, 6, and 9). Recently, another subgroup named CCD-like was found in some plant species such as *Malus domestica* and pepper [17,18]. The CCD gene family plays a vital role in different aspects of plants, including hormone biosynthesis, abiotic stress tolerance, plant growth and development, and color formation. For example, *Lilium brownii* CCD4 contributes to flower color variation via regulating the total carotenoid content [19]. The overexpression of *DcCCD4* in orange carrots causes their taproot color to turn pale yellow, with a sharp decrease in α- and β-carotene content [20]. OsCCD1 cleaves apocarotenoids to impact the pigmentation of Golden Rice 2 [21]. CsCCD1a plays a pivotal role in generating volatile β-ionone and pseudoionone from carotenoids [22]. As for strigolactone biosynthesis, CCD7 and CCD8 are two pivotal proteins that participate in its first stages [23,24]. Furthermore, by influencing the accumulation of endogenous ABA, PlNCEDs are involved in the seed dormancy of herbaceous peony [25]. And, in sweet potato, IbNCED1 is induced by ABA and GA3; the content of active GA3 is inhibited but accumulates ABA content when overexpressing *IbNCED1*, ultimately controlling the height and development of the sweet potato [26]. Moreover, AtNCED6 and AtNCED9 play key roles in ABA biosynthesis [27]. Further research has exhibited the significance of AtNCED3-regulated ABA biosynthesis under the mediation of AtNGA1 [28]. The silencing of *FaNCED1* in strawberry fruits results in a decrease in its ABA level and forms in uncolored fruits [29]. In addition, CCDs have been heterologously expressed in *Escherichia coli* and yeast. For instance, the introduction of *CsCCD1a* from *Camellia sinensis* in *E. coli* could generate β-ionone from carotenoids [22]. The expression of heterologous CCD1s from *Vitis vinifera* or *Petunia hybrida* in yeast is able to produce β-ionone [30]. In addition, different transcriptions factors have been investigated to modulate CCDs under different conditions. *Arabidopsis* MYB96 directly regulates *NCED2* and *NCED6* to modulate ABA biosynthesis [31]. WRKY57 can directly bind to the W-box of NCED3 and RD29A promoters to regulate the drought tolerance of *Arabidopsis* [32].

The CCD gene family has been widely reported in crops and horticultural plants, while there has been a lack of systematic CCD analysis in medicinal plants. *S. miltiorrhiza* is a well-known medicinal plant in the Lamiaceae family [33], which contributes to benefits in the cardiocerebral vascular system. In addition, *S. miltiorrhiza* is one of the model medicinal plants due to the knowledge of its whole genomic information, stable genetic transformation system, and abundant secondary metabolites, which can provide references for other medicinal plants [34]. In the present study, a total of 21 SmCCD genes have been genome-wide surveyed from *S. miltiorrhiza*, and the physicochemical characteristics including phylogeny, conserved motifs, gene structure, *cis*-elements, expression pattern, etc., were fully analyzed. This study may provide a foundation for future functional investigations of CCD genes in *S. miltiorrhiza* and other medicinal plants.

## 2. Results

### 2.1. Identification of CCD Genes Family Members

A total of 21 homologous CCD genes were excavated from the *S. miltiorrhiza* genome based on the HMM model for the REP65 domain (PF03055). Each CCD gene was renamed according to its orthologous relationship with *Arabidopsis* CCD members (Table S1). The fundamental physical and chemical properties of SmCCDs, including the length of the coding sequence, theoretical pI, protein molecular weight, aliphatic index, instability index and subcellular location, were analyzed (Table 1). The length of SmCCD proteins ranged from 432 to 645 amino acids (aa), the majority of them kept 450–580 aa, and SmCCD4d encoded the smallest one, while SmCCD7 was the largest. In addition, the protein molecular weight varied from 47.87 kDa to 72.15 kDa, and the pI varied between 4.88 and 8.67. As for the instability index, this ranged from 29.09 for SmCCD8 to 44.97 for SmCCD4f, including 7 proteins classified as unstable and 14 as stable. The subcellular localization prediction analysis of 21 SmCCD sequences showed that all of them were predicted in cytoplasm, chloroplast or both. Simultaneously, CCD4h, CCD4l, and CCD7 were also mitochondria-localized.

### 2.2. Phylogenetic Tree Analysis, Chromosome Locations, and Collinearity Analysis

**Figure 1:**
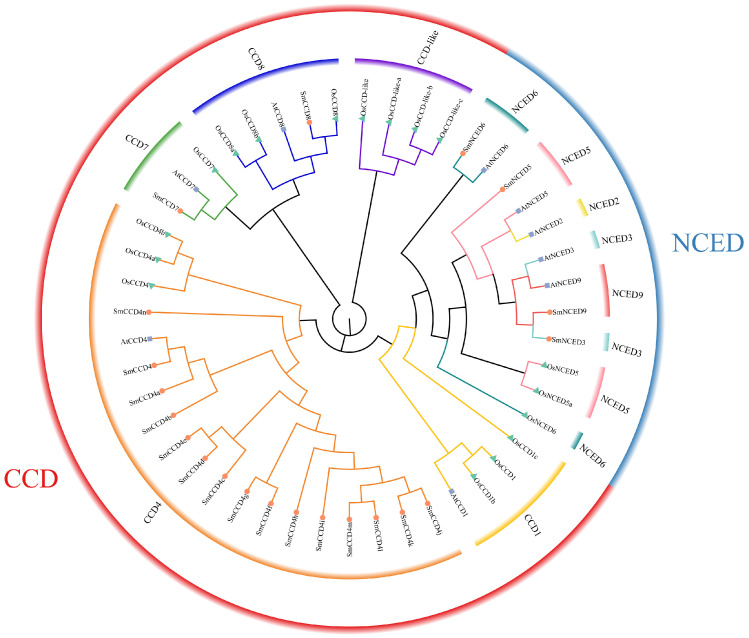
Phylogenetic tree demonstrating the evolutionary relationships of CCD proteins among *Arabidopsis*, rice, and *S. miltiorrhiza*. The maximum neighbor-joining (NJ) method tree was set with 1000 bootstrap replicates and applied to construct a phylogenetic tree with the MEGA11.0 software. The CCDs from *Arabidopsis*, rice, and *S. miltiorrhiza* were involved in CCD and NCED families. Different color denotes corresponding subfamilies.

To elucidate the phylogenetic relationships of SmCCD proteins, a neighbor-joining phylogenetic tree was constructed using CCD proteins distributed in *Arabidopsis* (9), rice (17), and *S. miltiorrhiza* (21) (Figure 1). The result revealed that 21 SmCCDs were classified into four subfamilies (CCD4, CCD7, CCD8, and NCED). A total of 15 SmCCDs were clustered into CCD4 subfamily (SmCCD4, SmCCD4a, SmCCD4b, SmCCD4c, SmCCD4d, SmCCD4e, SmCCD4f, SmCCD4g, SmCCD4h, SmCCD4i, SmCCD4j, SmCCD4k, SmCCD4l, SmCCD4m, and SmCCD4n), 4 members were grouped into NCED (SmNCED3, SmNCED5, SmNCED6, and SmNCED9), and only 1 member was clustered in CCD7 and CCD8, respectively. However, none of them was classified into the CCD1, NCED2, and CCD-like class. However, the OsCCD proteins were sorted into five subfamilies: CCD1, CCD4, CCD-like, CCD7, CCD8 and NCED. Among them, the CCD1, CCD4, CCD8, and NCED subfamilies each contained three members; while the CCD7 subfamily contained one member, the CCD-like subfamily contained four members. In addition, we found a closer genetic distance between AtCCDs and SmCCDs. The uneven distribution of SmCCDs may lead to the possibility of diversity and bias to achieve some function.

**Figure 2:**
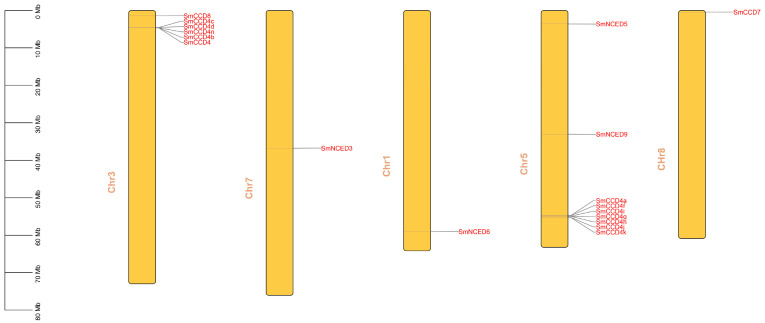
Chromosomal localization of SmCCD genes.

**Figure 3:**
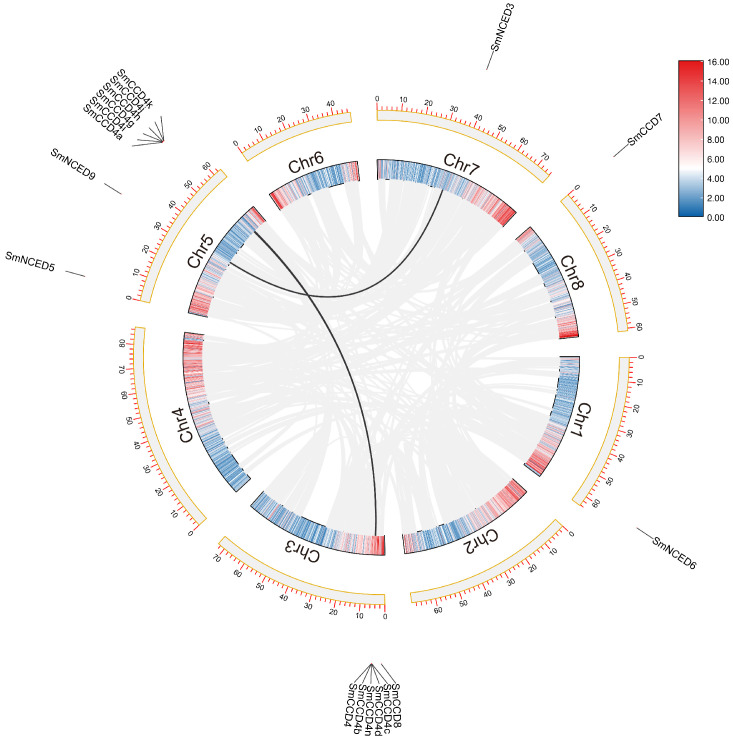
The intraspecific collinearity analysis of SmCCDs. Eight chromosomes have emerged using yellow frame with gray background rectangles. The homologous SmCCD genes were displayed using black curves. All genes with collinearity relationship were represented using gray curves.

The chromosome distribution showed that the SmCCDs were mainly located in five chromosomes (Figure 2), and the chromosomal distribution was uneven, with nine SmCCDs (42.9%) located on the Chr5, six SmCCDs located on the Chr3, and one SmCCD distributed between Chr1, Chr7, and Chr8, respectively. Furthermore, due to sequencing differences, the remaining three SmCCDs failed to obtain accurate location information. Intraspecific collinearity analysis was developed on the 21 SmCCD genes (Figure 3); 2 pairs of collinearity relationships were found, with SmNCED9 on Chr5 having a collinear gene on Chr7 and SmCCD4n on Chr3 having a collinear gene with SmCCD4k on Chr5, respectively, indicating that they may originate from segmental duplication. Furthermore, the collinearity analysis with *A. thaliana* and *O. sativa* demonstrated a certain degree of close relations with some collinear genes (Figure S1).

### 2.3. Motif Analysis and Cis-Element Analysis

**Figure 4:**
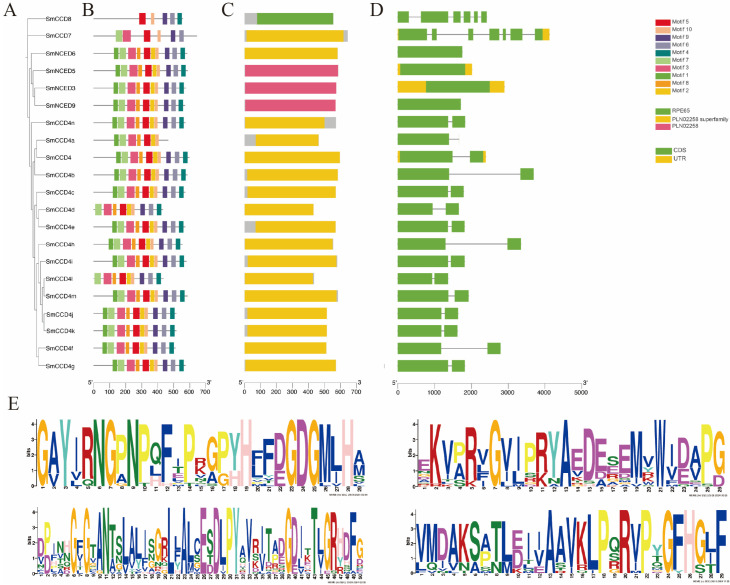
Phylogenetic relationship, motif, and gene structure analysis of SmCCDs. (**A**) Phylogenetic tree of SmCCDs. (**B**) Motif analysis achieved by MEME software. Rectangles with different colors represent corresponding motifs. (**C**) Conserved domain analysis of SmCCDs. Green rectangles indicate PLN02258 superfamily regions, yellow rectangles indicate RPE65 domain regions, and PLN02258 is shown using pink rectangles. (**D**) Gene structure of SmCCDs. Green rectangles indicate CDS regions, yellow rectangles indicate UTR regions, and introns are shown using black lines. (**E**) Protein sequences of motifs 1, 2, 3, and 4.

The gene structures uncovered that SmCCD members varyingly possessed one to seven exons and zero to six introns. The size of the different exons also showed obvious differences between them simultaneously. Among them, SmCCD7 had the most abundant exons and introns, while SmNCED3, SmNCED5, SmNCED6, and SmNCED9 lacked introns. Furthermore, only four members, namely SmNCED3, SmNCED5, SmCCD4, and SmCCD7, contained an untranslated region (UTR). Then, the MEME tool contributed to the analysis of the features of the SmCCD motifs, with a sum of 10 motifs being analyzed (Figure 4). Interestingly, most of the SmCCD members contained all 10 motifs except SmCCD4a, SmCCD4d, SmCCD4l, SmCCD7, and SmCCD8. In addition, the motif numbers of SmCCD8 were far lower than others, suggesting that it could be unique or important in physiological process. In conclusion, these results also proved the accuracy of the SmCCDs’ phylogenetic clustering to a certain extent.

**Figure 5:**
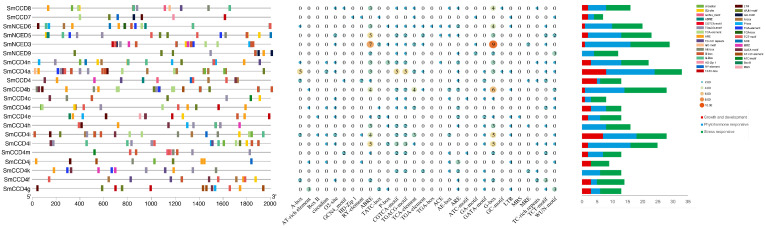
Analysis of *cis*-elements in the promoter of SmCCD genes.

To further explore the potential function and regulation of SmCCD genes, the *cis*-acting elements in the promoters of SmCCDs were analyzed using PlantCARE (Figure 5). We identified that hormones, along with defense and stress, light-responsive, and specific-expression elements and so on are found in the promoter region. In addition, 15 SmCCD genes contained an ABA-responsive element, and SmNCED3 possessed the most. Furthermore, plant growth primarily relies on the process of endosperm development, meristem development, and circadian rhythm, and the corresponding elements can also be found on most promoters, displaying the possible relationship between SmCCDs and plant development.

### 2.4. Prediction of Three-Dimensional (3D) Structure of SmCCDs

**Figure 6:**
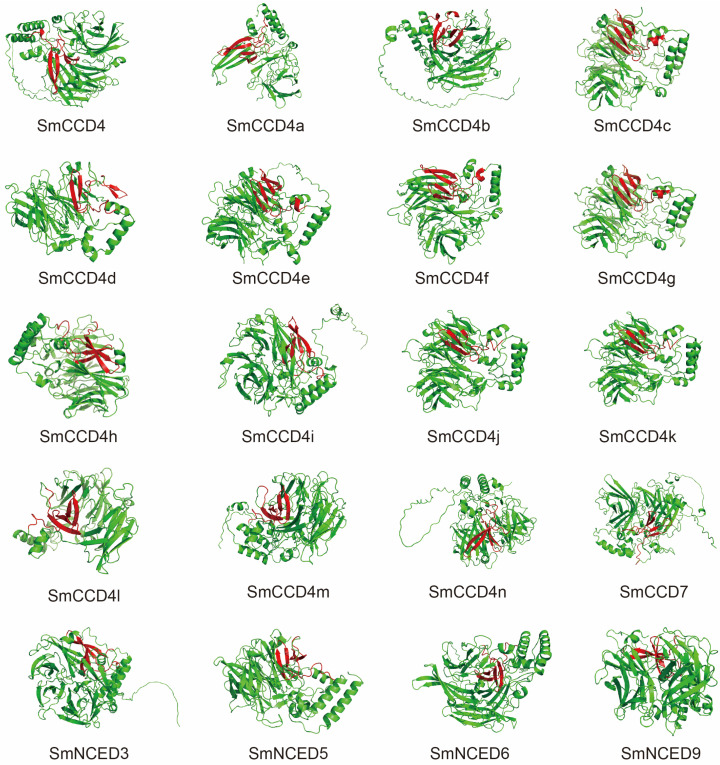
Protein tertiary structure of SmCCDs. The red part of the structure is the conserved domain of motif 1 from Figure 4E.

According to the prediction of the CCD protein’s tertiary structure (Figure 6), these mainly consisted of β-strands and random coils. Moreover, except for SmCCD8, the other CCD members all contained a conserved domain composed of one or two β-sheets, and some of them, such as SmCCD4, SmCCD4b, SmCCD4c, SmCCD4e, SmCCD4g, and SmCCD4f, also had a α-helix in this domain. Unlike SmCCD4s, NCED and other CCD groups exhibited lower α-helixes, which comprised the main differences between them.

### 2.5. Protein Interaction Network Analysis

**Figure 7:**
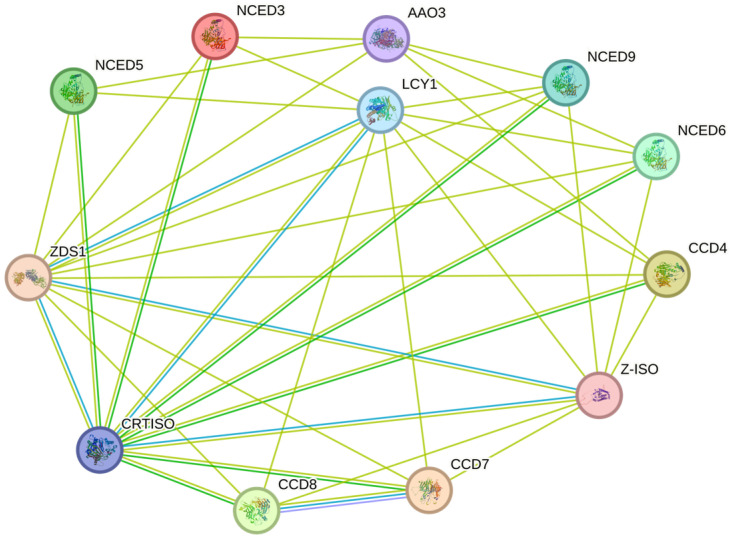
Analysis of SmCCD protein interaction network in *S. miltiorrhiza*.

Interaction networks could potentially be a powerful tool for clarifying their own function and involvement in some pathways or biological reaction process. Therefore, the String database served as a candidate choice to preliminarily prove the importance of the interaction network. The CCD proteins in *S. miltiorrhiza* corresponded to the orthologous proteins in *Arabidopsis* (Figure 7). It is noteworthy that some members, such as CCD8 and CCD7, interacted with each other, indicating that they may be involved in the same signal pathway or development process via forming complex compounds. Moreover, some factors like receptors or enzymes related to ABA synthesis and signals like AAO3 and carotenoid biosynthesis-related genes such as LCY1, ZDS1, Z-ISO, etc., appeared in the network, indicating the pivotal and irreplaceable roles of CCDs in ABA metabolism and other related physiological process. In general, the identification of the SmCCD interaction network is crucial for improving practical regulation mechanisms. Due to a great portion of genes being clustered into the CCD4 branch, an interaction network surrounded by CCD4 was constructed. Except for ABA signal-relevant proteins, a massive number of proteins are involved in carotenoid biosynthesis, while the carotenoid pathway is also one of the important ways to synthesize ABA. Moreover, CCD4 is deduced to interact with DXS (Figure S2), suggesting the potential ability to regulate the synthesis of terpenoids, such as tanshinones. Overall, CCD4 may play a key role in the regulation of secondary metabolite biosynthesis.

### 2.6. Expression Patterns of SmCCDs in Different Tissues, Stress Condition, and the Screening of Candidate Functional SmCCD Genes

**Figure 8:**
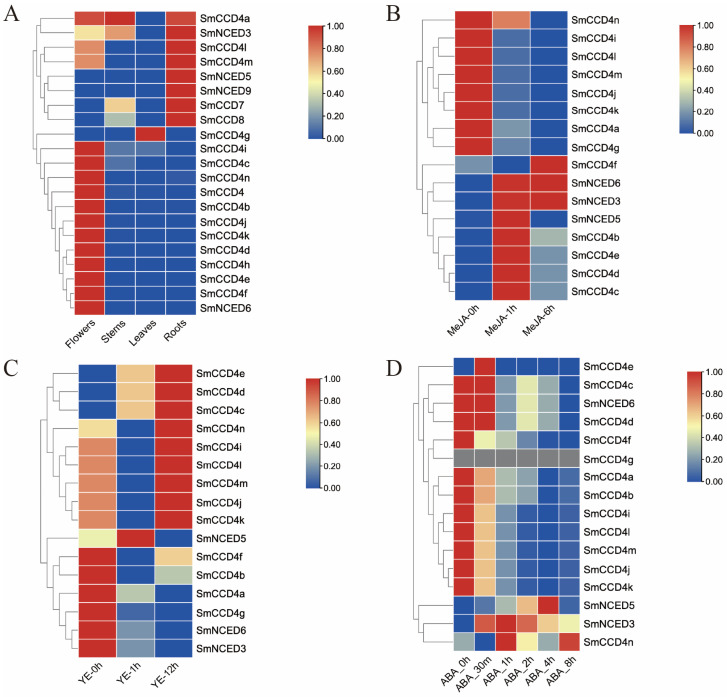
Heatmap of the *SmCCD* gene expression profiles in various tissues and treatments. (**A**) Heatmap of the SmCCD gene expression profiles in roots, stems, leaves, and flowers. (**B**) Heatmap of the SmCCD gene expression profiles under MeJA treatment. (**C**) Heatmap of the SmCCD gene expression profiles under YE treatment. (**D**) Heatmap of the SmCCD gene expression profiles under ABA treatment. The colored scale on the right represents the relative expression level, and red is used to suggest a high expression level while blue is the opposite.

The expression patterns of the SmCCD genes in different tissues (roots, stems, leaves, and flowers) and stress conditions (MeJA, YE, and ABA treatment) were analyzed on the basis of RNA-seq data (Figure 8). Tissue-specific analysis revealed that *SmCCD4a*, *SmNCED3*, *SmCCD4l*, *SmCCD4m*, *SmNCED5*, *SmNCED9*, *SmCCD7*, and *SmCCD8* were expressed highly in the roots, and may participate in the biosynthesis of secondary metabolites in *S. miltiorrhiza*. *SmCCD4g* showed more abundance in leaves, and other SmCCD4 members, together with *SmNCED6*, were expressed more highly in flowers.

It has been verified that MeJA, ABA, and YE treatments are efficient in enhancing tanshinones and/or phenolic acids in *S. miltiorrhiza* hairy roots. Here, the expression analysis demonstrated that *SmCCD4f*, *SmNCED6*, *SmNCED3*, *SmNCED5*, *SmCCD4b*, *SmCCD4e*, *SmCCD4d*, and *SmCCD4c* were MeJA-responsive, in which SmCCD4f expression peaked when treated after 6h. Furthermore, *SmCCD4e*, *SmCCD4d*, *SmCCD4c*, *SmCCD4n*, *SmCCD4i*, *SmCCD4l*, *SmCCD4m*, *SmCCD4j*, *SmCCD4k*, and *SmNCED5* showed higher expression levels when treated with YE. Furthermore, the expression of *SmCCD4n*, *SmNCED5*, and *SmNCED3* showed a higher expression under ABA treatment in a later stage. In summary, the results revealed that the expression of *SmCCD4e* and *SmNCED5* was activated under the aforementioned treatment, indicating that they may play a role in different response pathways.

To further detect the function of SmCCDs, fluridone-treated *S. miltiorrhiza* seedlings were prepared, and samples induced for 0, 1, and 12 h were harvested to perform transcriptome sequencing. Three cDNA libraries containing 52128240, 53397642, and 45649718 raw reads were constructed, with an average GC content of 50.04% and a Q30 value of over 95.42%. A total of nine unigenes were annotated as CCDs, of which two (TRINITY_DN4200_c0_g1 and TRINITY_DN4928_c1_g1) could be found in the fluridone transcriptome; TRINITY_DN4200_c0_g1 was determined only be significant in the down-regulated expression at 1 h, which confirmed it as SmCCD4. Therefore, it may play a potential role in ABA biosynthesis (Table S2, Figure S3).

## 3. Discussion

CCDs are an ancient but small family commonly found in plants, and are involved in catalyzing carotenoids to other compounds such as plant hormones, ultimately forming aromatic compounds [35]. The CCD family contributes to various physiological processes, including color formation, growth, and development, as well as tolerance to abiotic stress [36]. At present, the CCD gene family has been identified in different plants: 9 plants in *Arabidopsis* [37], 30 in *Brassica napus* [38], 10 in cucumber [39], and 23 in *Populus trichocarpa* [40]. In this study, a total of 21 SmCCD genes were identified in the *S. miltiorrhiza* genome and classified into four subfamilies (SmCCD4, 7, 8, and NCED) in view of the phylogenetic relationships with *Arabidopsis* and rice. The CCD1 and CCD-like subfamily were not detected in *S. miltiorrhiza*, suggesting the specific evolution of CCDs across various plant species. Among them, the CCD4 class contained fifteen SmCCD members, while CCD7 and CCD8 contained only one and four were found in the NCED subfamily, indicating that CCD4 genes might produce function differentiation and redundancy and proceed with gene expansion in the process of evolution, leading to a function exploration of SmCCD4s. A multiple sequence alignment showed that all the SmCCD members commonly harbor the conserved RPE65 domain. Notably, all NCED members in *S. miltiorrhiza* lacked introns which was similar to NCEDs in other plants [41,42]; this may be due to a precise post-transcriptional process when facing stress conditions. Furthermore, the SmCCD genes are unevenly distributed between five chromosomes. For example, nine SmCCDs (42.9%) were located on Chr5, six SmCCDs were located on Chr3, and one SmCCD was distributed on Chr1, Chr7, and Chr8, respectively. Several CCD gene members are closely located on the same chromosome, indicating that they may occur in segmental duplication, which was similar to poplar CCDs distribution [40]. And for tertiary structure prediction, compared to NCED proteins, CCDs had more diverse structures, but there is no redundancy in other proteins. Moreover, it can be deduced that the SmCCD4 subfamily members are highly structurally similar, and based on the theory that the conserved His residues determine the enzymatic activity of CCD proteins [15], the SmCCD4 subfamily could also be reasonably inferred as a group of functionally active proteins. From the perspective of structure, CCD4 usually exhibited more structural variation compared to the other CCD genes; this may be one of the causes of the high abundance of CCD4s in *S. miltiorrhiza*.

In order to excavate function possibilities for SmCCDs, the expression patterns of tissue-specific and abiotic stress responses were analyzed using the corresponding transcriptome database. The candidate SmCCD genes were expressed differentially in the examined tissues, including in the roots, stems, leaves, and flowers. For example, SmCCD4 members (*SmCCD4a*, *SmCCD4l*, and *SmCCD4m*), *SmCCD7*, *SmCCD8*, and SmNCEDs (*SmNCED5*, *SmNCED9*) showed more abundance in roots, implying that they may be involved in the biosynthesis of secondary metabolites such as tanshinones in *S. miltiorrhiza*. Most of SmCCD4 members, including *SmCCD4*, were more abundant in flowers, indicating their role in color formation. For example, the expression levels of *CCD4* in both pale-yellow and white petals were high, which was related to the high degradation activity of carotenoids, ultimately influencing the color of *Eustoma grandiflorum* flowers [43]. CCD members not only have a high tissue-specific pattern but also respond to various abiotic stresses. For instance, the CCD genes of melon responded to abiotic stress to different degrees [44]. *LcNCED2*, *LcCCD1*, and *LcCCD2* genes demonstrated a positive correlation with increasing tolerance ability to drought stress [45]. CCD4 in sweet potato was selected by dramatically responding to salt and dehydration stress, and further heterologous expression in *Arabidopsis* proved that lbCCD4 weakened its salt tolerance [46]. Most promoters of SmCCDs contained massive cis-regulatory elements related to responding to abiotic and biotic stress, which provided preliminary evidence that various stress conditions could regulate CCD genes. In this study, elicitation treatments such as MeJA, YE, and ABA were utilized to treat *S. miltiorrhiza* hairy roots and enhance the content of bioactive compounds. For instance, the addition of 100 μM MeJA to *S. miltiorrhiza* hairy roots improved the tanshinone and phenolic acid production. The application of YE in *S. miltiorrhiza* cultures was effective in accumulating tanshinones [47]. An ABA-responsive element was found in most SmCCD promoters, and a MeJA-responsive element appeared in promoters of SmCCD4k, SmCCD4h, SmCCD4, and SmNCED3, suggesting the potential function of SmCCDs in hormone signaling response. And the expression pattern of the SmCCD genes showed that SmCCD4 members (*SmCCD4f*, *SmCCD4b*, *SmCCD4e*, *SmCCD4d*, and *SmCCD4c*) and SmNCEDs (*SmNCED6*, *SmNCED3*, and *SmNCED5*) were induced by MeJA treatment. Most SmCCD4s showed higher expression levels when treated with YE. The abundances of *SmCCD4e*, *SmCCD4n*, *SmNCED5*, and *SmNCED3* were elevated when treated by ABA. For example, AtNCED3 encoded a rate-limiting enzyme as a candidate gene for stress-induced ABA accumulation in *Arabidopsis* [48]. And ABA RNA-seq demonstrated that SmNCED3 peaked highest after 1 h of ABA treatments. Furthermore, the prediction of the protein interaction network of SmCCDs hinted towards the possible potential connection with the ABA signaling pathway and carotenoid pathway. For example, *CsNCED3*, *CsCCD1*, and *CsCCD4* were activated by CsERF061 to accumulate carotenoid content further and were accompanied by a variation in ABA concentration [49], providing possibilities for a regulatory module associated with CCD to regulate carotenoids or even the ABA pathway. Therefore, the NCED/CCD genes are not only conserved in the ABA biosynthesis pathway, but they are also highly correlated with abiotic stress response. However, further study is needed to determine whether SmNCEDs were indeed involved in ABA synthesis.

For the functional excavation of the SmCCD4 subfamily, it could be observed that all the SmCCD4 subfamily members only have one intron, which is different from the other CCD members in *S. miltiorrhiza*, while the NCED lacked any introns; it may be speculated that NCEDs evolved from CCD4 [50]. Moreover, the NCEDs were considered as rate-limiting enzymes in ABA biosynthesis. Previous studies have exhibited the potential of CCD4 for abiotic stress response. For instance, when treated with 200 mg/L of ABA, the expression level of several genes involved in carotenoids metabolism including CCD4 increased, contributing to the accumulation of total carotenoids content [51]. In apples, MdCCD4b and MdCCD4c were obviously up-regulated when exposed to plant hormones [52]. Moreover, RNAi-CCD4 in potato exhibited an increase in carotenoid content in its tubers and the appearance of new phenotypes due to heat sprouting, which may be a pivotal reason for the tuber response to heat [14]. We may have also potentially found that the expression of *SmCCD4* decreased when treated with fluridone. Additionally, similar to some key enzymes in the phenolic acid and tanshinone biosynthetic pathway such as PAL, TAT, C4H, RAS, CYP98A14, GGPPS, the expression level of *SmCCD4* varied under ABA treatment and responded the strongest 2 h after ABA treatment [53]. Previous studies have proved that CCD4 promoted the formation of xanthoxin and then further metabolized to ABA [54]. Furthermore, based on the conclusions that have been studied, it is reasonable to assume that SmCCD4 may act as a regulator of the abundance of precursors in ABA biosynthesis. Overall, this comprehensive analysis of the CCD gene family in *S. miltiorrhiza* shed new light onto the characteristics of SmCCD genes and also provided references for other medicinal plants.

## 4. Materials and Methods

### 4.1. Identification of CCD Genes Family Members in S. miltiorrhiza

To identify the CCD genes in *S. miltiorrhiza,* the genome file was achieved from the laboratory resource bank [55]. The sequences of CCD in *A. thaliana* were acquired from the TAIR database (https://www.arabidopsis.org/ (accessed on 12 September 2024)). Further, the sequence information of *Oryza sativa* was made available from the Rice Genome Annotation Project (http://rice.plantbiology.msu.edu/ (accessed on 12 September 2024)). The conserved domain RPE65 (PF03055) was obtained from InterPro (https://www.ebi.ac.uk/interpro/scan.html/ (accessed on 12 September 2024)). PF03055 was used to identify candidate CCD proteins in *S. miltiorrhiza* using the HMMER3.0 software with E-value ≤ 10^−5^ [44]. NCBI CD-search was further employed to confirm the integrity of the RPE65 domain (https://www.ncbi.nlm.nih.gov/Structure/bwrpsb/bwrpsb.cgi (accessed on 12 September 2024)). The *S. miltiorrhiza* CCD genes were named according to their homology with the ones in *A. thaliana*. The physical and chemical properties such as the length, molecular weight, theoretical pI, and the instability index of CCD proteins were predicted from ExPASy (https://web.expasy.org/protparam/ (accessed on 12 September 2024)). The subcellular localization of the CCD proteins was predicted through iPSORT (https://ipsort.hgc.jp/#predict (accessed on 12 September 2024)) and Plant-mPLoc predictor (http://www.csbio.sjtu.edu.cn/bioinf/plant-multi/ (accessed on 12 September 2024)).

### 4.2. Phylogenetic Tree Analysis, Chromosome Locations, and Collinearity Analysis of SmCCDs

The CCD protein sequences from *A. thaliana*, *O. sativa*, and *S. miltiorrhiza* were employed to analyze the phylogenetic relationships. The phylogenetic tree was constructed with MEGA11 software using the maximum neighbor-joining (NJ) method tree with 1000 bootstrap replicates. The chromosome location of the CCD genes was analyzed in the *S. miltiorrhiza* genome via TBtools2.0 [56], and then a visualization of the chromosome locations was made using the MCScanX v3.9 tool [57]. TBtools was applied to visualize the collinearity relationship.

### 4.3. Motif Analysis and Cis-Element Analysis

The diversity of the motifs in the SmCCD proteins was evaluated through the MEME 5.5.7 online forecasting site (https://meme-suite.org/meme/ (accessed on 12 September 2024)); the number of motifs was set to 10, whereas other conventional parameters were set to default. To excavate the potential capacity that was responsive to environmental stress, a 2000 bp genomic sequence upstream of the translation initiation codon (ATG) of each SmCCD gene was extracted using TBtools and the cis-regulatory elements were further evaluated using the PlantCARE online website (http://bioinformatics.psb.ugent.be/webtools/plantcare/html/ (accessed on 12 September 2024)).

### 4.4. Prediction of Secondary and Three-Dimensional (3D) Structure of SmCCDs

The SWISS-MODEL (https://swissmodel.expasy.org (accessed on 12 September 2024)) website was utilized to construct 3D structures of the SmCCD proteins and then the models were visualized using the PyMOL3.0 program.

### 4.5. Protein Interaction Network of SmCCDs

We utilized the Orthovenn2 website to seek orthologous genes between *A. thaliana* and *S. miltiorrhiza* (https://orthovenn2.bioinfotoolkits.net/home (accessed on 12 September 2024)). Then, the String10 website (https://cn.string-db.org/ (accessed on 12 September 2024)) was utilized to forecast, with the high confidence set to 0.70, the protein interaction network of the AtCCD proteins and to draw the protein network of the SmCCD proteins. Finally, the drawing and landscaping of the protein interaction network were achieved.

### 4.6. Expression Patterns of SmCCDs in Different Tissues and Elicitation

The expression patterns of each SmCCD gene were analyzed under different elicitor treatments including MeJA, YE, and ABA, which was conducted in our lab [58]. Moreover, the various tissues, including flowers, stems, leaves, and roots, were used to analyze the tissue expression profile of SmCCD genes.

### 4.7. Pre-Experiment for CCD Screening, Transcriptome Sequencing and Analysis

The seeds of *S. miltiorrhiza* were sowed and germinated in nutrient soil, when the fourth true leaves appeared on the plants, the seedlings were transferred to pots with nutrient soil. After a one-month growth period in a greenhouse under conditions of 50% relative humidity, 16 h light/8 h darkness, and a constant temperature of 25 °C, the seedlings were prepared for a follow-up experiment. For the fluridone treatments, the seedlings were sprayed with a final concentration of 50 μM, which was obtained from macklin (Shanghai, China). All the samples were put in liquid nitrogen instantly and stored in a −80 °C refrigerator for further use.

The total RNA isolated from the seedlings and RNA quality or purity was assessed by Nanodrop (Thermo Fisher Scientific (Waltham, MA, USA)). The Messenger RNA was purified using the polyA selection method with oligo_(dT)_ beads. The fragmentation of mRNA synthesized the first strand of cDNA, and the second strand of cDNA that was synthesized occured after the addition of dNTPs, RNase H, and DNA polymerase I, this was then purified with AMPure XP beads. The process of fragmentation was carried out in an Illumina proprietary fragmentation buffer. The cDNA libraries were composed of a screened fragment selection with AMPure XP beads after PCR amplification. Depending on Illumina HiSeqTM2500 and PE100, the cDNA library was sequenced [59]. Majorbio (Shanghai, China) carried out the sequence assembly and data analysis of RNA-seq.

## 5. Conclusions

In this study, a sum of 21 SmCCD genes were identified from the *S. miltiorrhiza* genome database. Simultaneously, the characteristics of SmCCDs displayed similarities and differences. Phylogenetic tree analysis revealed that SmCCD members were grouped into four subfamilies, namely SmCCD4, 7, 8, and NCED. And these SmCCDs localized in five chromosomes. Furthermore, conserved motifs, the prediction of subcellular localization, collinearity analysis, *cis*-elements, and protein interaction were also conducted. Moreover, the relatively low level of collinearity may contribute to a divergence in function. The expression level of SmCCD genes varied under different hormone treatment, suggesting that they may be involved in various signaling pathways and metabolic regulation. SmCCD4 may play a pivotal role in ABA biosynthesis. These results may lay a solid foundation for the further functional excavation of the SmCCD gene family.

## Figures and Tables

**Table 1:** Physiochemical properties of the SmCCDs.

Name	Number of Amino Acids	Molecular Weight/kDa	Theoretical pI	Instability Index	Aliphatic Index	Subcellular Localization
SmCCD4	596	65.44	6.64	36.34	77.42	Chloroplast, Cytoplasm
SmCCD4a	464	49.99	7.31	36.06	90.41	Chloroplast, Cytoplasm
SmCCD4b	584	64.06	6.36	41.02	85.36	Chloroplast, Cytoplasm
SmCCD4c	571	62.16	5.95	37.01	84.20	Chloroplast, Cytoplasm
SmCCD4d	432	48.07	5.59	29.09	93.63	Cytoplasm
SmCCD4e	570	62.25	6.79	34.29	88.14	Chloroplast, Cytoplasm
SmCCD4f	511	55.63	5.91	34.45	83.78	Cytoplasm
SmCCD4g	572	62.12	6.29	36.13	87.19	Chloroplast, Cytoplasm
SmCCD4h	554	61.04	6.03	37.18	80.61	Mitochondria, Chloroplast, Cytoplasm
SmCCD4i	579	64.18	6.85	36.23	90.6	Cytoplasm
SmCCD4j	515	56.79	5.63	36.54	88.04	Chloroplast, Cytoplasm
SmCCD4k	515	56.78	5.84	38.38	89.01	Cytoplasm
SmCCD4l	435	47.87	4.88	34.43	90.32	Mitochondria, Chloroplast, Cytoplasm
SmCCD4m	585	64.24	5.47	44.66	87.50	Chloroplast, Cytoplasm
SmCCD4n	572	62.74	6.41	34.71	84.90	Chloroplast, Cytoplasm
SmCCD7	645	72.15	8.67	42.39	78.19	Mitochondria, Chloroplast, Cytoplasm
SmCCD8	556	62.21	6.56	39.95	78.38	Chloroplast, Cytoplasm
SmNCED3	574	63.87	6.24	41.97	78.01	Cytoplasm
SmNCED5	586	65.03	6.15	43.65	77.20	Cytoplasm
SmNCED6	583	64.33	5.89	42.28	88.27	Chloroplast, Cytoplasm
SmNCED9	569	63.02	7.23	44.97	84.85	Chloroplast, Cytoplasm

## Data Availability

Data are contained within the article.

## Supplementary Materials

The following supporting information can be downloaded at https://www.mdpi.com/article/10.3390/ijms252313138/s1.
