# Supplementary material for: Genome-Wide Identification and Expression Analysis of Carotenoid Cleavage Dioxygenase Genes in Salvia miltiorrhiza
Source: Int J Mol Sci. 2024 Dec 6;25(23):13138. doi: 10.3390/ijms252313138 (PMC11641897; doi:10.3390/ijms252313138)
Supplement: Supplementary file 1 [file ijms-25-13138-s001.zip › ijms-3307613 - supplementary.pdf]

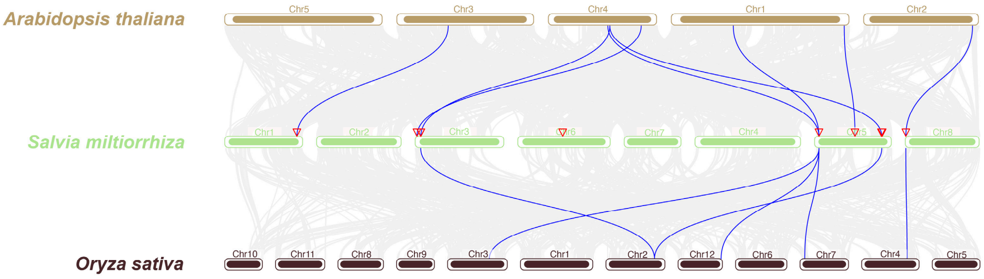

Figure S1. Collinearity analysis between *S. miltiorrhiza*, *A. thaliana and O. sativa*.

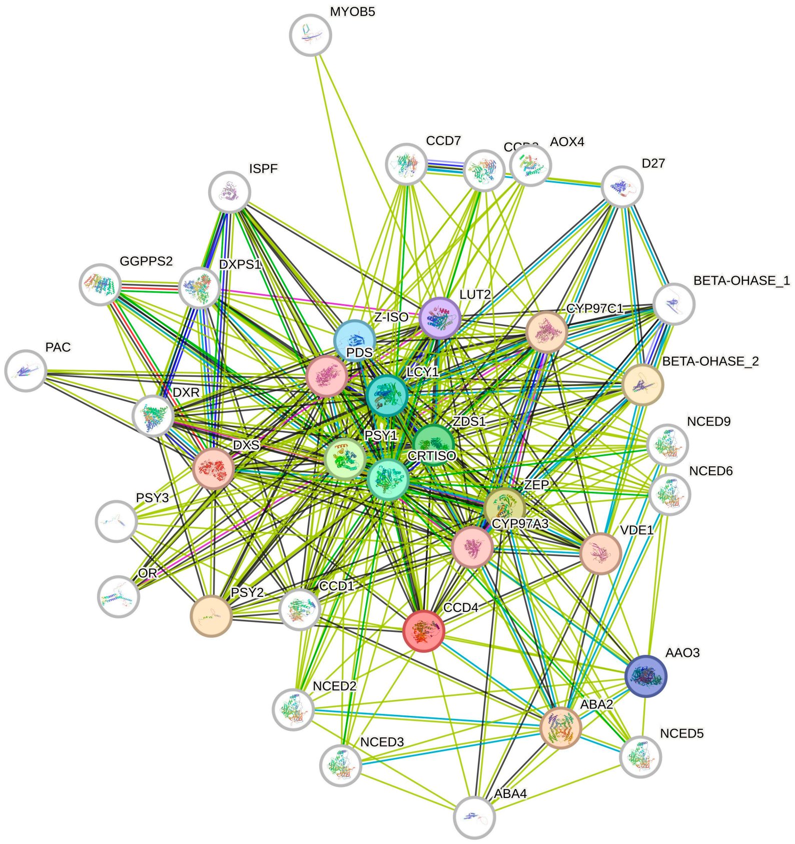

Figure S2. Analysis of SmCCD4 protein interaction network in *S. miltiorrhiza*.

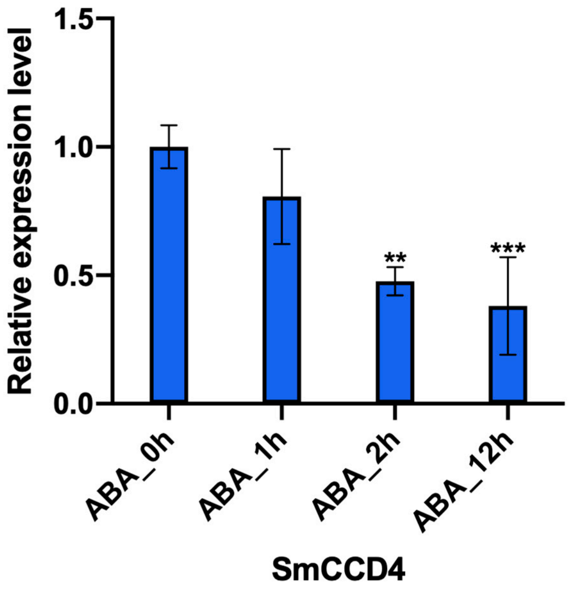

Figure S3. The qPCR validation for SmCCD4 under ABA treatment. ** $p < 0.01$; *** $p < 0.001$.
